# Phylogenetic landscape of Monkeypox Virus (MPV) during the early outbreak in New York City, 2022

**DOI:** 10.1080/22221751.2023.2192830

**Published:** 2023-04-17

**Authors:** Luz H. Patiño, Susana Guerra, Marina Muñoz, Nicolas Luna, Keith Farrugia, Adriana van de Guchte, Zain Khalil, Ana Silvia Gonzalez-Reiche, Matthew M. Hernandez, Radhika Banu, Paras Shrestha, Bernadette Liggayu, Adolfo Firpo Betancourt, David Reich, Carlos Cordon-Cardo, Randy Albrecht, Rebecca Pearl, Viviana Simon, Aria Rooker, Emilia Mia Sordillo, Harm van Bakel, Adolfo García-Sastre, Dusan Bogunovic, Gustavo Palacios, Alberto Paniz Mondolfi, Juan David Ramírez

**Affiliations:** a Department of Pathology, Molecular, and Cell-Based Medicine, Icahn School of Medicine at Mount Sinai, New York, NY, USA; b Department of Preventive Medicine, Public Health and Microbiology, Universidad Autónoma de Madrid, Madrid, Spain; c Facultad de Ciencias Naturales, Centro de Investigaciones en Microbiología y Biotecnología-UR (CIMBIUR), Universidad del Rosario, Bogotá, Colombia; d Department of Genetics and Genomic Sciences, Icahn School of Medicine at Mount Sinai, New York, NY, USA; e Department of Microbiology, Icahn School of Medicine at Mount Sinai, New York, NY, USA; f Global Health and Emerging Pathogens Institute, Icahn School of Medicine at Mount Sinai, New York, NY, USA; g Division of Infectious Diseases, Department of Medicine, Icahn School of Medicine at Mount Sinai, New York, NY, USA; h Center for Vaccine Research and Pandemic Preparedness (C-VARPP), Icahn School of Medicine at Mount Sinai, New York, NY, USA; i Icahn Genomics Institute, Icahn School of Medicine at Mount Sinai, New York, NY, USA; j The Tisch Cancer Institute, Icahn School of Medicine at Mount Sinai, New York, NY, USA; k Department of Microbiology, Centre for Inborn Errors of Immunity, Precision Immunology Institute, Icahn School of Medicine at Mount Sinai, New York, NY, USA

**Keywords:** Monkeypox virus, cell culture, genome sequencing, mutations, Phylogenetic analysis, Orthologous Poxvirus Genes (OPG)

## Abstract

Monkeypox (MPOX) is a zoonotic disease endemic to regions of Central/Western Africa. The geographic endemicity of MPV has expanded, broadening the human-monkeypox virus interface and its potential for spillover. Since May 2022, a large multi-country MPV outbreak with no proven links to endemic countries has originated in Europe and has rapidly expanded around the globe, setting off genomic surveillance efforts. Here, we conducted a genomic analysis of 23 MPV-infected patients from New York City during the early outbreak, assessing the phylogenetic relationship of these strains against publicly available MPV genomes. Additionally, we compared the genomic sequences of clinical isolates versus culture-passaged samples from a subset of samples. Phylogenetic analysis revealed that MPV genomes included in this study cluster within the B.1 lineage (Clade IIb), with some of the samples displaying further differentiation into five different sub-lineages of B.1. Mutational analysis revealed 55 non-synonymous polymorphisms throughout the genome, with some of these mutations located in critical regions required for viral multiplication, structural and assembly functions, as well as the target region for antiviral treatment. In addition, we identified a large majority of polymorphisms associated with GA > AA and TC > TT nucleotide replacements, suggesting the action of human APOBEC3 enzyme. A comparison between clinical isolates and cell culture-passaged samples failed to reveal any difference. Our results provide a first glance at the mutational landscape of early MPV-2022 (B.1) circulating strains in NYC.

## Introduction

The Monkeypox virus (MPV), an Orthopoxvirus that causes the zoonotic disease Monkeypox (MPOX), is closely related to the Variola virus, the causative agent of smallpox, and the most clinically relevant member of the Poxviridae family until its eradication due to vaccination [1]. Since its discovery in 1958 during an outbreak of vesicular illness in captive monkeys shipped to Copenhagen and the first reported infection in a human patient on 1 September 1970, in the Democratic Republic of Congo, an increase in the number of human cases has been confirmed across 15 different countries throughout Central and West Africa [2].

Historically, MPV has been divided into two different clades (Central and West African). However, a new clade (clade 3) emerged as a consequence of the current outbreak outside Africa. Since its identification, multiple lineages have been characterized within clade 3 (B.1, A.1.1, A.1, and A.2), suggesting that evolutionary events may have occurred throughout the current outbreak, allowing not only diversification and dispersion of the virus to other geographical areas outside Africa but also its adaptation to new hosts across new countries [3]. Lineage B.1, diverged from A.1 lineage (2018–2019 outbreak), and is considered to be closely related to the current outbreak of human monkeypox [4]. Phylogenetic analysis reveals that B.1 lineage segregates in a divergent phylogenetic branch, thus generating several clusters (sub-lineages) such as B.1.1, B.1.2, B.1.3, B.1.4, B.1.5, B.1.6, B.1.7, and B.1.8, which have been identified in different geographical regions, suggesting ongoing viral evolution and spread [5].

Such features coupled with other factors such as increasing trends towards urbanization, deforestation, and human displacement due to civil wars and armed conflicts [6], have allowed geographic endemicity of MPV to expand exponentially, hence broadening the human-monkeypox virus interface and its potential for spillover. In addition, species-to-species boundaries, have been threatened as an inadvertent consequence of global commerce and travel, leading to an expansion outside traditional virus hosts and novel risks for epizootic outbreaks [7]. Such was the case for the 2003 United States multistate outbreak of Monkeypox that affected over 70 people in the states of Illinois, Indiana, Kansas, Missouri, Ohio and Wisconsin in 2003. At that time, trace back investigations confirmed that all human cases were associated to contact with prairie dogs (*Cynomys* species) which had been infected by other African rodents imported from Ghana during their captivity, lodging and/or transportation [8]. Subsequently, in 2018, imported cases of human-to-human transmission were confirmed both in the United Kingdom and in Israel, affecting primarily returning travelers from Nigeria and a healthcare worker caring for one of the UK patients, and later in Singapore [9], providing irrefutable evidence for human-to-human transmission. This was supported by previous studies from the Congo basin where enhanced human-to-human transmission suggested that transmission efficiency of the virus might also be increasing [10,11].

Since May 2022, a large multi-country MPV outbreak with no proven links to endemic countries originated in Europe and has rapidly spread around the globe. Following the initial cryptic transmission event, most transmissions appear to have occurred through specific human-to-human networks primarily among men who have sex with men, suggesting potential human adaptation and a change in transmission dynamics of the virus. As of 1 February 2023, the Centers for Disease Control and Prevention (CDC) has confirmed 85,702 MPV cases distributed across 110 different countries worldwide, 30,123 of which have been identified in the USA [12]. Since the initial reported case in Massachusetts, in a returning traveler from Canada on 17 May 2022, a total of 30,123 cases have been confirmed, with New York being one of the epicenters of the MPV outbreaks in the US with 4222 reported cases.

Genomic surveillance was shown to be a powerful tool for understanding disease dynamics both during the COVID-19 pandemic, and in the ongoing MPV outbreak [4,13,14]. Thus far, more than 2000 outbreak-related MPV genomes have been sequenced (https://www.epicov.org/epi3/frontend#cb2ea), which have allowed the rapid reconstruction and phylogenomic characterization of the virus, as well as the identification of mutational bias (From GA to AA or TC to TT) driven by apolipoprotein B mRNA editing catalytic polypeptide-like 3 (APOBEC3), as signal potential MPV human adaptation in ongoing microevolution [4,15]. APOBEC3 enzymes are cytidine deaminases that act on the single strand DNA during the replication or transcription inducing mutagenesis. In response to a viral infection, these enzymes can be upregulated, thus inhibiting a wide range of viruses [4].

However, in some cases, the APOBEC3-mediating mutations fail to completely destroy the virus, generating viable viruses but with altered characteristics such as immune escape or reduced pathogenicity, which could facilitate the cryptic transmission of virus in the populations, as have been observed in MPV [15].

MPV genomes have been sequenced and characterized globally, enabling assessment of their genomic diversity, evolutionary trajectory, and phenotypic characteristics [4,14,16]. However, limited information is available about the genomic behaviour of this virus in New York [17].

On the other hand, several studies have assessed the genomic behaviour of viruses maintained in cultures, especially RNA viruses. Some of these studies have revealed adaptive changes after serial viral passages in vitro, but none earlier than four passages [18,19]. Others have entirely failed to document differences at a genomic level when comparing viruses from directly collected samples and in-vitro grown viruses [20]. To date, knowledge about the adaptive mutations that could emerge during cell culture and whether this could provide insight into functional aspects of infection is scarce.

Taking into account these considerations, this study mainly focuses on analyzing the genomic characteristics of 23 MPV-positive samples from New York City during the early outbreak. Additionally, it aims to compare the genomic sequences of clinical isolates with those of culture-passaged samples.

## Materials and methods

### Samples

**Figure 1.**
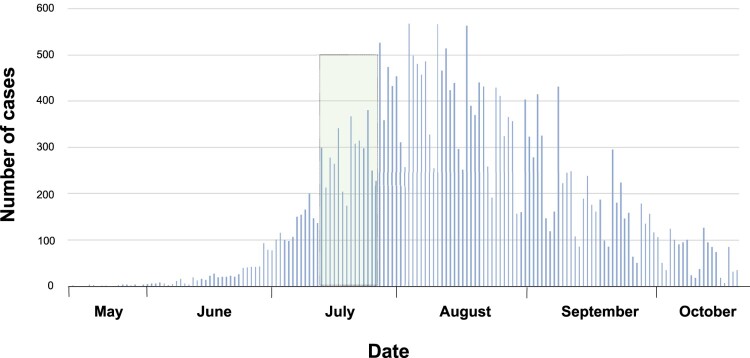
Number of cases MPV reported through time in U.S. The graphic refers 27,632 cases reported of MPV by May to October, 2022 in U.S. The green colour represents the sampling period.

A total of 23 MPV samples positive by real-time polymerase chain reaction (qPCR), tested at the Molecular Microbiology Laboratories of the Icahn School of Medicine at Mount Sinai, were included for whole genome sequencing. Samples were collected between 11 July and 24 July 2022 (Figure 1). Eleven specimens were directly collected from skin lesions (comprising nine swabs included in VTM and two dry swabs in sterile containers) and twelve that were isolated from cell culture. Additionally, a paired analysis between six direct and cultured samples was performed. Regrettably, not all samples (clinical and culture-passaged samples) could be compared in this study, due to either difficulties in isolating the virus during cell-culture and/or to low quality of sequencing. The metadata of samples and the Ct value from clinical samples are summarized in Table 1.

**Table 1.** Data of the samples subjected to sequencing analysis.

Sample ID	Transport medium	Sample origin	RT-PCR Ct Value	Total # of generated Monkeypox reads	Read length (bp)	Mean depth of coverage	Completeness (%)	Clade	Lineage
PV67599	DRY	CS	15.71	38,542	150	22.5	92.8	IIb	B.1
PV67601	DRY / VTM	CS	16.43 / 18.68	286,104	150	168.9	100.0	IIb	B.1.2
PV67603	DRY / VTM	CS	24.54 / 19.02	262,248	150	157.1	100.0	IIb	B.1
PV67604	DRY / VTM	CS	19.68 / 21.57	736,352	150	438.9	100.0	IIb	B.1.7
PV67606	DRY	CPS	NA	217,164	150	135.8	100.0	IIb	B.1
PV67610	DRY	CPS	NA	50,396	150	30.0	99.3	IIb	B.1.3
PV67611	VTM	CPS	NA	81,522	150	50.4	99.9	IIb	B.1.2
PV67614	DRY	CPS	NA	100,516	150	61.8	100.0	IIb	B.1.2
PV67617-CS	DRY / VTM	CS	16.38 / 18.84	310,120	150	184.0	100.0	IIb	B.1
PV67617-CPS	NA	CPS	NA	138,740	150	86.0	99.9	IIb	B.1
PV67619-CS	DRY / VTM	CS	11.34 / 14.13	102,698	150	59.9	100.0	IIb	B.1.8
PV67619-CPS	NA	CPS	NA	79,212	150	48.7	99.9	IIb	B.1.8
PV67620-CS	VTM	CS	17.38	86,092	150	51.6	99.6	IIb	B.1.1
PV67620-CPS	NA	CPS	NA	193,332	150	121.0	100.0	IIb	B.1.1
PV67621-CS	DRY / VTM	CS	18.59 / 17.89	166,784	150	98.8	100.0	IIb	B.1.3
PV67621-CPS	NA	CPS	NA	190,652	150	118.7	100.0	IIb	B.1.3
PV67622-CS	VTM	CS	20.26	56,304	150	32.9	93.2	IIb	B.1.2
PV67622-CPS	NA	CPS	NA	111,266	150	69.7	99.9	IIb	B.1.2
PV67624	VTM	CS	15.79	101,034	150	60.2	99.9	IIb	B.1.7
PV67625	VTM	CPS	NA	318,518	150	198.5	100.0	IIb	B.1.7
PV67626-CS	DRY	CS	18.87	39,318	150	22.9	97.4	IIb	B.1
PV67626-CPS	NA	CPS	NA	1,283,846	150	813.3	100.0	IIb	B.1
PV67629	DRY	CPS	NA	45,342	150	27.5	97.5	IIb	B.1.7

CS: Clinical sample. CPS: Culture passaged samples.

### Virus isolation and propagation

Six samples from MPV positive patients underwent in vitro cell culture by infecting human cells (Human telomerase reverse transcriptase (hTERT)-immortalized primary cells from Dussan lab [21]). After 2 days, all infected cultures showed a clear cytopathic effect (CPE) typical of Orthopoxviruses. All work with viral cultures was performed in the BSL-3 Conventional Biocontainment F facility at ISMMS by trained personnel using standard operating procedures approved by the Mount Sinai Institutional Biosafety Committee.

### DNA extraction

Dry swabs were resuspended in 1 mL of 50% ChemagicTM lysis buffer and vortexed for 30 s. Meanwhile, VTM-collected swabs underwent gentle shaking (200 rpm, 30 min, at room temperature). Additionally, supernatant from hTERT-infected cells (48 hpi) was collected for DNA extraction, while supernatants from uninfected cells were used as a control. A 300 µl aliquot was transferred from each specimen to individual wells of a 2 mL deep-well plate. Subsequently, 300μL of lysis buffer and extraction master mix (4μL Poly(A) + 10 μl Proteinase K) were added to each well. The DNA from MPV-CS and MPV-Culture was extracted using the ChemagicTM Viral DNA/RNA 300 Kit H96 (CMG-1033-S; PerkinElmer) on the automated ChemagicTM 360 instrument (2024-0020; PerkinElmer) following the manufacturer's protocol and as previously described (Perkin Elmer, n.d.).

### Genome sequencing and assembly

Paired-end Nextera XT (Illumina, cat. FC-131-1096) libraries were prepared from 1 ng of total DNA and were sequenced on a MiSeq instrument with 2 × 150 bp reads. Monkeypox genomes were assembled using a custom reference-based pipeline, as previously described [22], using ON563414 as the reference genome. For clinical samples, we initially processed and assembled the DRY and VTM samples separately and verified that their genotypes matched. In cases where we obtained genomes from more than one specimen, we ensured that the variant patterns were consistent for both specimens. We then combined the sequencing data across specimens to maximize genome coverage and produce the final genomes. In Table 1, we have indicated the samples for which we combined the data for the assemblies by marking them as “DRY/VTM” in the “Transport Medium” column. The number of reads, coverage, and completeness obtained for each sample during the sequencing and assembly are described in Table 1.

### Phylogenetic analysis and genetic diversity

A total of 651 complete and high-quality MPV genomes from different regions of the world, available in the GISAID database (www.gisaid.org) and the National Center for Biotechnology Information (NCBI), were used to evaluate the phylogenomic relationships. These genomes were last accessed on 31 August 2022, and their metadata is summarized in Table S1.

To evaluate the phylogenetic relationships, the obtained sequences were aligned against the reference sequence of the current human MPV outbreak (hMPXV-1) MPV-M5312_HM12_Rivers (Lineage A from clade IIb; GenBank accession number: NC_063383.1) using the NextClade tool v1.5.4 (https://clades.nextstrain.org/). The Maximum-likelihood (ML) tree was reconstructed using IQtree v.2.1.3 [23], and the bootstrap analysis was performed with 1000 iterations. The tree was exported in Newick format and visualized and edited using the Interactive Tree Of Life V4 (http://itol.embl.de).

To identify nucleotide variations, the 23 genomes included in this study were compared with the reference genome NC_063383.1 using the NextClade tool v1.5.4. All detected mutations were carefully inspected using the Integrative Genomics Viewer software. Finally, snipit (https://github.com/aineniamh/snipit) was used to extract and visualize variant sites potentially compatible with APOBEC3-mediated viral genome editing from sequence alignments.

## Results

### Sequencing statistics

We obtained and sequenced amplicons using the Illumina MiSeq system, which generated a variable number of reads ranging between 38,542 and 1,283,846. The average read length was 150 bp, with a depth of coverage ranging between 22.5X to 198.5X and completeness ranging between 92.8% to 100% (Table 1).

### Phylogenetic inference

**Figure 2.**
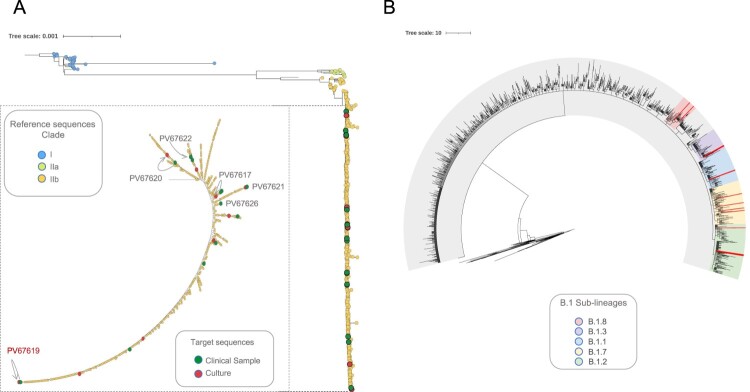
Phylogenetic relationship of MPV genomes in the global context. (A) Maximum likelihood tree from whole genome SNPs for the 674 genomes sequences analyzed. Each colour represent the three different clusters of MPV described so far (I, IIa and IIb). A magnification of the cluster IIb (to visualize the 23 genomes included in this study) was included. The green dots represent the samples from patients and the red dots the samples from cell culture. The figure shows the identification of each paired sample with their respective connection between direct sample and culture (lines) and highlight in red whose paired sample with differences between them. (B) Maximum likelihood tree highlighting the sub-lineages B.1 identified in this study. Each colour into the tree represents a different sub-lineage; the red lines show the position of the 16 genomes mapped into five different sub-lineages B.1.

Phylogenetic analysis revealed the presence of three distinct clades (Clade I, IIa and IIb) in agreement with the genetic population structure globally recognized for MPV. Of the total 674 genome-sequences analyzed, 626 (including the 23 genomes from New York analyzed in this study) from the ongoing MPV B.1 multi-country outbreak, clustered into Lineage B.1 (clade IIb) (Figure 2). Furthermore, when assessing location of these 23 genomes throughout the phylogenetic tree, we observed that seven mapped into Lineage B.1 and 16 mapped into five different sub-lineages of B.1 (B.1.1, B.1.2, B.1.3, B.1.7 and B.1.8) (Figure 2, Table 1, Table S2). No difference in the lineage assignment was observed when comparing the six-paired samples (clinical samples (CS) vs. culture-passaged samples (CPS)) (Figure 2, Table 1).

### Nucleotide diversity

**Figure 3.**
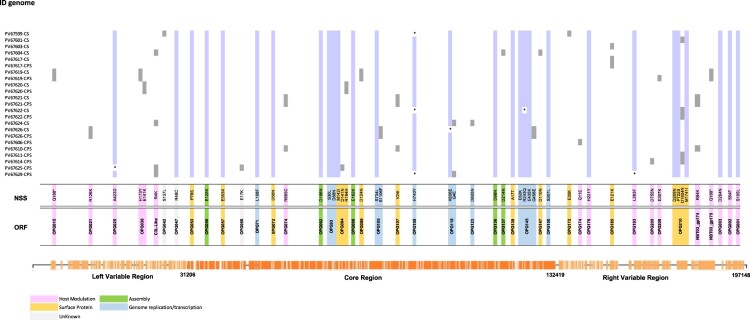
SNP characterization between 23 genomes analyzed. Single nucleotide polymorphisms (SNP) found in the 23 MPV isolates (Genome ID) compared with the reference sequence (NC_063383.1). The purple colour represents the non-synonymous substitution (NSS) shared in more than 90% of genomes analyzed, and the grey colour the NSS shared between CS-CPS samples or found in just some genomes.* represents the identification of a N in that position. The colours located in the bottom of the figure represent the function in which have been associated the ORF.

**Figure 4.**
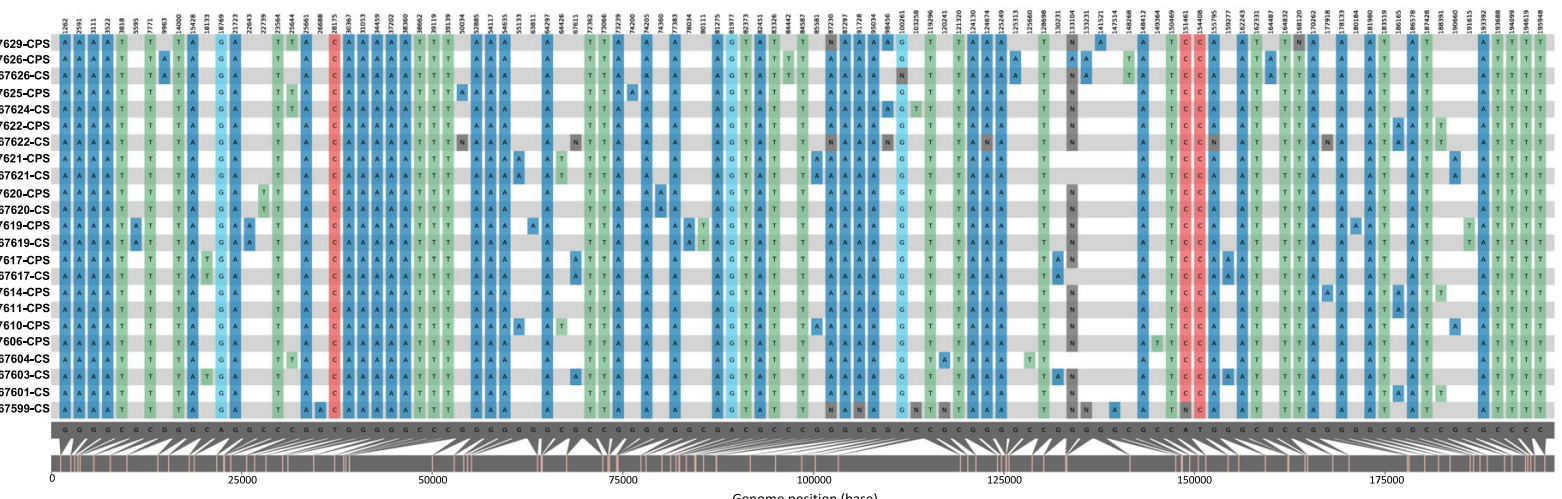
Nucleotide diversity between 23 genomes analyzed. The colours represent the SNPs found throughout MPV genome, to compare the 23 samples included in the study with the reference sequence (NC_063383.1). We observed a major representation in the GA > AA and TC > TT substitutions.

Mapping for specific mutations revealed 104 polymorphic sites that distinguished the 23 NY MPV genomes from the reference genome (NC_063383.1). 55/104 (53%) identified as non-synonymous mutations, 29 of them (53%) shared in more than 90% of genomes evaluated and 26 identified in just some genomes (Figure 3). The 55 non-synonymous mutations were observed across 43 ORFs, 23 of them distributed in the core region, 13 through the right variable region (OPG172, OPG174, OPG176, OPG185, OPG193, OPG205, OPG208, OPG210, NBT03_gp174, NBT03_gp175, OPG003, OPG002 and OPG001) and 7 through left variable region (OPG015, OPG021, OPG025, OPG038, C3L-Like, OPG42 and OPG047) (Figure 3, Table S3). In addition, 98 (hyper) mutation signatures including GA > AA and TC > TT; G > A (58%); and C > T (35%) nucleotide replacements were observed (Figure 4), suggesting a mutational bias driven by APOBEC3 enzymatic activity. Of the 98 (hyper) mutation signatures identified, 61 were shared among the 23 MPV genomes included in this study and 37 were identified in only some of the genomes (Figure 4) (25 identified as non-synonymous mutations and 12 as synonymous mutations (Table S4)). Finally, we observed that from 37 (hyper) mutation signatures, eight were unique and shared within the B.1.1, B.1.2, B.1.3 and B.1.7 sub-lineages. Two mutations were identified in sub-lineage B.1.1 (C22739T:OPG038 and G74360A:OPG094), one in sub-lineages B.1.2 and B.1.7 (G186165A:OPG210 and C25644T:C3L-Like, respectively); and four mutations sub-lineage B.1.3 (G55133A:OPG074, C64426T:OPG083, G85581A:OPG107 and G190660A:NBT03_gp174) (Table 2).

**Table 2.** SNPs identified with GA > AA and TC > TT nucleotide replacements shared between the different lineages.

Lineage	OPG	Position	Ref	Alt	AA Change ALT	Function
B.1.1	OPG038	22739	C	T	E141K	NFKB Inhibitor
	OPG094	74360	G	A	R194H	Myristylated protein
B.1.2	OPG210	186165	G	A	D1604N	Surface glycoprotein
B.1.3	OPG074	55133	G	A	R665C	Activates of ERK1/2 signaling pathway
	OPG083	64426	C	T	Synonymous variant	Serine protease
	OPG107	85581	G	A	V24I	Entry-fusion complex
	NBT03_gp174	190660	G	A	R84K	Brix domain protein
B.1.7	C3L-like	25644	C	T	R4K	Prevents complement activation

## Discussion

The increasing number of human MPOX cases over the last few decades, along with its expanding geographic distribution, including its extension to non-endemic regions, has raised significant concerns regarding the ongoing adaptability, cross-species transmission, infectivity, and transmissibility of the MPV. Furthermore, the rapid worldwide dissemination of MPV during the 2022 multi-country outbreak, and its genetic link to the 2017–2018 Nigerian outbreak cluster, suggest that the virus's spread has been a silent ongoing phenomenon, in which human-to-human transmission has been influenced by evolutionary dynamics and viral adaptation to the human host [11]. Recent reports highlight cases of MPOX co-infection with other viruses, such as VZV (Varicella-Zoster Virus) [24], SARS-CoV-2 [25], and HIV (Human Immunodeficiency Virus) [26,27]. Interestingly, according to the WHO External Situation Report 14 (as of January 19, 2023), 48% of the reported MPOX cases have occurred in HIV-positive patients [28]. Such co-infections, mainly in the HIV/AIDS population, could lead to increased vulnerability and risk of developing severe and complicated forms of the disease, placing MPOX as a new player in the roster of HIV-related opportunistic infections.

The MPV double-stranded DNA genome comprises 196.858 bp [29] (∼ 197 kb) encoding for approximately 190 genes [30]. Its genomic architecture includes a central (core) region which is relatively conserved and known to encode for important replication and assembly function proteins [30], as well as two variable (right and left) regions which have been linked to its broad host range and pathogenic determinants [29,30] as predicted based on genome-wide comparisons. Other components of the genome architecture such as non-coding regions and inverted terminal repeats (ITRs), along with fluctuations in gene content due to insertions/deletions may account for enhanced adaptability, transmissibility [31] and possibly ongoing evolution of the 2022 MPV outbreak lineage.

We analyzed whole-genome MPV sequences from patients in NYC and compared paired viral genomic sequences from swabs and cultures. Our aim was to determine the mutational profile, locations, and novel acquired mutations from early circulating lineages/clades and their likely influence on diverse aspects of viral biology. The genomic data from the 23 New York City samples analyzed in our study are consistent with other reports that demonstrate the following: (i) clustering of current MPV 2022-outbreak circulating strains within the B.1 lineage (Clade IIb) and (ii) the occurrence of further and ongoing differentiation onto B.1. sub-lineages (Figure 2). Our results confirm, as suggested by other authors [4,11], that the ongoing multi-country outbreak appears to trace back to a single origin, and that the B.1 lineage is diversifying further while accumulating an increasing number of mutations, potentially reflecting an accelerated adaptive mechanism to the human host, as previously proposed [5].

Although Orthopoxviruses are known to have lower rate of nucleotide substitutions (1–2 substitutions per genome per year) [32], we identified 55 non-synonymous mutations among the 23 MPV genomes from New York analyzed when compared against the reference genome. Isidro *et al* previously reported that the 2022 MPV strain diverged approximately 6-to-12 times more than previously estimated for other Orthopoxviruses, with an average of 50 SNPs compared to its related 2018–2019 strains, thus signaling a continuous selection pressure and accelerated evolution [4].

Upon close examination against the reference genome [NC_063383.1], we observed that across 43 ORFs, 53% (23) of these mutations were distributed across the core region, followed by 30% (13) mutations spread through the right variable region, and 16% (7) in the left variable region (Figure 3, Table S3). The fact that the majority of these mutations are located in the relatively conserved core region of the genome may be a sign of enhanced selective pressure, as one would expect as the virus further adapts to humans. Among the proteins located in the core region, we highlight those specifically involved in viral replication, such as OPG071, a DNA polymerase, OPG098 (L4R), a nucleic acid binding protein, OPG105 (J6R), a DNA-dependent RNA polymerase, and OPG145 (A18R), a DNA helicase. In addition, essential structural and assembly proteins, including OPG092 (G7L) and OPG136 (A10L), a virion core protein, also revealed variations. Some of these variations may explain, in part, the effects on more efficient viral replication (OPG071) or predict an adverse response to MPV-specific treatment, as is the case for OPG057, the homologous protein to F13L in Vaccinia virus, recognized to be a putative target of the currently used antiviral tecovirimat [33].

Additional mutations were identified in the right and left variable regions of the examined genomes. These mutations predominantly affected proteins with immune-related functions, such as OPG001 (C23L), a chemokine binding protein that binds to host CC chemokines (beta chemokines) like RANTES [34] (https://www.uniprot.org/uniprotkb/Q805H7/entry), OPG002 (C22L), a Crm-B secreted TNF-alpha-receptor-like protein, and OPG210 (B21R), an immunogenic surface glycoprotein [14,35] (Figure 3).

Our findings on the mutational landscape of early MPV-2022 (B.1) circulating strains in New York City provide further insights into how genetic changes may affect virulence, human-to-human transmission, vaccine escape, and possibly influence treatment resistance. In this context, previous findings by Chen et al. highlighted mutations in five genes that may affect function [36] or play a role in the inactivation of OPG153 through a frameshift mutation, ultimately leading to an increase in virus replication levels [37].

On the other hand, recent studies suggest that APOBEC3 enzyme activity might be a potential driver for virus adaptive evolution of the currently circulating B.1 lineage [4,15]. Our findings are aligned with previous studies which have reported hypermutation signatures (extensive and inactivating mutations on strand DNA produced by APOBEC3) (Figure 4), suggestive of APOBEC3 enzyme activity. Among the hypermutation signatures found in our samples, we highlight those located in genes encoding proteins involved in the interaction with the host immune system, replication, and viral structure (Table S4), some of which are shared within the different B.1 sub-lineages (Table 2). Considering that APOBEC3 enzyme presents antiviral activity against viruses, the mutations driven by this enzyme could reduce the pathogenicity and symptoms caused by MPV infections, facilitating not only viral transmission but also triggering possible MPV adaptive evolution, as previously suggested [4,14,15,38]. Another interesting finding in our study was the increased number of APOBEC3-driver mutations identified when compared with the 46 initial mutations described by Isidro et al. [4]. These findings lend further support to the possibility of APOBEC3-associated adaptive mutations occurring as part of the adaptive evolution of the circulating MPV strains most recently shown.

We further aimed to investigate the effects of cell culture passaging on original clinical samples to assess possible adaptive effects on virulence, transmissibility, immune-related functions, mutation dynamics, and how potential mutations may influence gene function. When contrasting genomic sequences of MPV derived from patient swabs (wild-type) with those from cell cultures, we did not observe any differences in the number and positions of substitutions throughout the genome (Figure 3). This observation is consistent with findings from previous studies, in which mutational adaptive changes are usually not recorded until after at least four passages in vitro. This phenomenon has been described for RNA viruses [18,19], as well as some DNA viruses, including poxviruses, such as Fowlpox virus [20]

We consider that conducting deeper sampling by increasing the number of passages in vitro, as well as using different cellular lines, would provide valuable insights into aspects such as cell tropism, viral replication kinetics, and the characterization of mutational profiles associated with selection pressures. Moreover, investigating the effects of passaging on specific clinical scenarios can help us better understand the implications of these findings.

Further genomic surveillance studies are needed to assess the evolution of current MPV circulating strains and whether newly acquired mutations could have contributed to the rapid and efficient transmission of the virus during the 2022 multi-country outbreak and its expansion to non-endemic regions.

## Supplementary Material

Supplemental Material
